# A Screen for Genetic Suppressor Elements of Hepatitis C Virus Identifies a Supercharged Protein Inhibitor of Viral Replication

**DOI:** 10.1371/journal.pone.0084022

**Published:** 2013-12-31

**Authors:** Rudo L. Simeon, Zhilei Chen

**Affiliations:** 1 Artie McFerrin Department of Chemical Engineering, Texas A&M University, College Station, Texas, United States of America; 2 Department of Microbial and Molecular Pathogenesis, Texas A&M Health Science Center, College Station, Texas, United States of America; Scripps Research Institute, United States of America

## Abstract

Genetic suppressor elements (GSEs) are biomolecules derived from a gene or genome of interest that act as transdominant inhibitors of biological functions presumably by disruption of critical biological interfaces. We exploited a cell death reporter cell line for hepatitis C virus (HCV) infection, n4mBid, to develop an iterative selection/enrichment strategy for the identification of anti-HCV GSEs. Using this approach, a library of fragments of an HCV genome was screened for sequences that suppress HCV infection. A 244 amino acid gene fragment, B1, was strongly enriched after 5 rounds of selection. B1 derives from a single-base frameshift of the enhanced green fluorescent protein (eGFP) which was used as a filler during fragment cloning. B1 has a very high net positive charge of 43 at neutral pH and a high charge-to-mass (kDa) ratio of 1.5. We show that B1 expression specifically inhibits HCV replication. In addition, five highly positively charged B1 fragments produced from progressive truncation at the C-terminus all retain the ability to inhibit HCV, suggesting that a high positive charge, rather than a particular motif in B1, likely accounts for B1’s anti-HCV activity. Another supercharged protein, +36GFP, was also found to strongly inhibit HCV replication when added to cells at the time of infection. This study reports a new methodology for HCV inhibitor screening and points to the anti-HCV potential of positively charged proteins/peptides.

## Introduction

Hepatitis C virus (HCV) is a single-stranded, enveloped, positive-sense RNA virus of the *Flaviviridae* family [1]. HCV RNA polymerase exhibits a high mutation rate, causing the virus to exist as a quasispecies in a single patient [2]. Six major genotypes and numerous subtypes of HCV have been identified around the world [3]. HCV infection afflicts over 180 million people worldwide [4] and is the leading cause of cirrhosis and cancer of the liver [5], [6]. HCV-induced end-stage liver disease is the leading indication for liver transplantation in developed countries [7]. Until recently the only approved HCV therapy involved a 24 or 48 week regimen of combination therapy using pegylated interferon alpha and ribavirin [8], [9]. Interferon alpha-ribavirin mono-treatment is costly, time-consuming and riddled with serious and debilitating side effects such as depression, fatigue and flu-like symptoms [10], [11], resulting in many patients being unable to complete the therapy. In addition, interferon α-ribavirin therapy yields a sustained virological response (SVR) in only 50% of treated patients infected with the most common genotype [12]. Recent pharmacological advances have led to the development and approval of two new drugs, boceprevir and telaprevir, which greatly improve the treatment response to up to 79% of the patients [13], [14]. However, molecules that target specific viral proteins, including boceprevir, telaprevir and most of those in advanced clinical development, tend to foster drug-resistant variants [15], [16].

Genetic suppressor elements (GSEs) are short, biologically active gene fragments derived from a gene or genome of interest that act as transdominant inhibitors of biological functions [17], [18]. GSEs can exert their inhibitory effect through expressed antisense RNAs, structural RNAs, or peptide/protein fragments that bind to and disrupt critical biological interfaces. Screens or selections for GSEs typically do not require any previous knowledge of target gene(s)/protein(s) or the type of inhibitor (antisense RNAs, RNA decoys or transdominant mutants) that will most potently suppress the function of a specific gene. This feature of GSE screens/selections has empowered the approach to identify previously unknown viral genes that are essential for the infectious cycle of bacteriophage lambda [19]. Thus, the performance of GSE screens/selections has the potential to uncover new biological information even in a very thoroughly investigated system. Other successes of GSE selection include the elucidation of human immunodeficiency virus type 1 (HIV-1) latency [17], bovine viral diarrhea virus entry [20], tumor suppressor genes [21], genes that mediate cellular sensitivity to anticancer drugs [22], [23], regulators of transcription [24], and potential anticancer [25] and antiviral [26] targets.

In addition to their role as tools for studying viruses, GSEs are potential therapeutic agents. Some GSEs have been found to decrease viral loads of bovine viral diarrhea virus (BVDV) by 100- to 1000-fold [20], a potency on par with some of the most potent BVDV antiviral candidates in preclinical and clinical trials [27]. Even if the GSEs themselves are not ideal drugs, the molecules can serve as templates for the creation of small molecule mimetics, which can in turn be used as antivirals.

In this work we aimed to identify GSEs with anti-HCV activity. Using a hepatoma cell line, n4mBid, that reports HCV infection by a cell-death phenotype. Specifically, we developed an iterative selection strategy which gradually enriches anti-HCV genetic fragments that confer resistance to HCV-induced cell death. Surprisingly, the most strongly enriched element, a genetic element we named B1, is a 244 amino acid protein derived from a frame shifted enhanced green fluorescent protein (eGFP) [28] that was used as a filler during library cloning. B1 has a high net positive charge of 43 at pH 7, leading to a charge-to-molecular-weight ratio of 1.5. B1 also possesses strong ability to deliver protein/nucleic acid cargo into the mammalian cell cytosol [29]. In this work, we show that B1 inhibits HCV replication when expressed intracellularly and the inhibitory effect is largely mediated by its high overall charge.

### Results

#### GSE screens to identify genes involved in HCV infection

**Figure 1:**
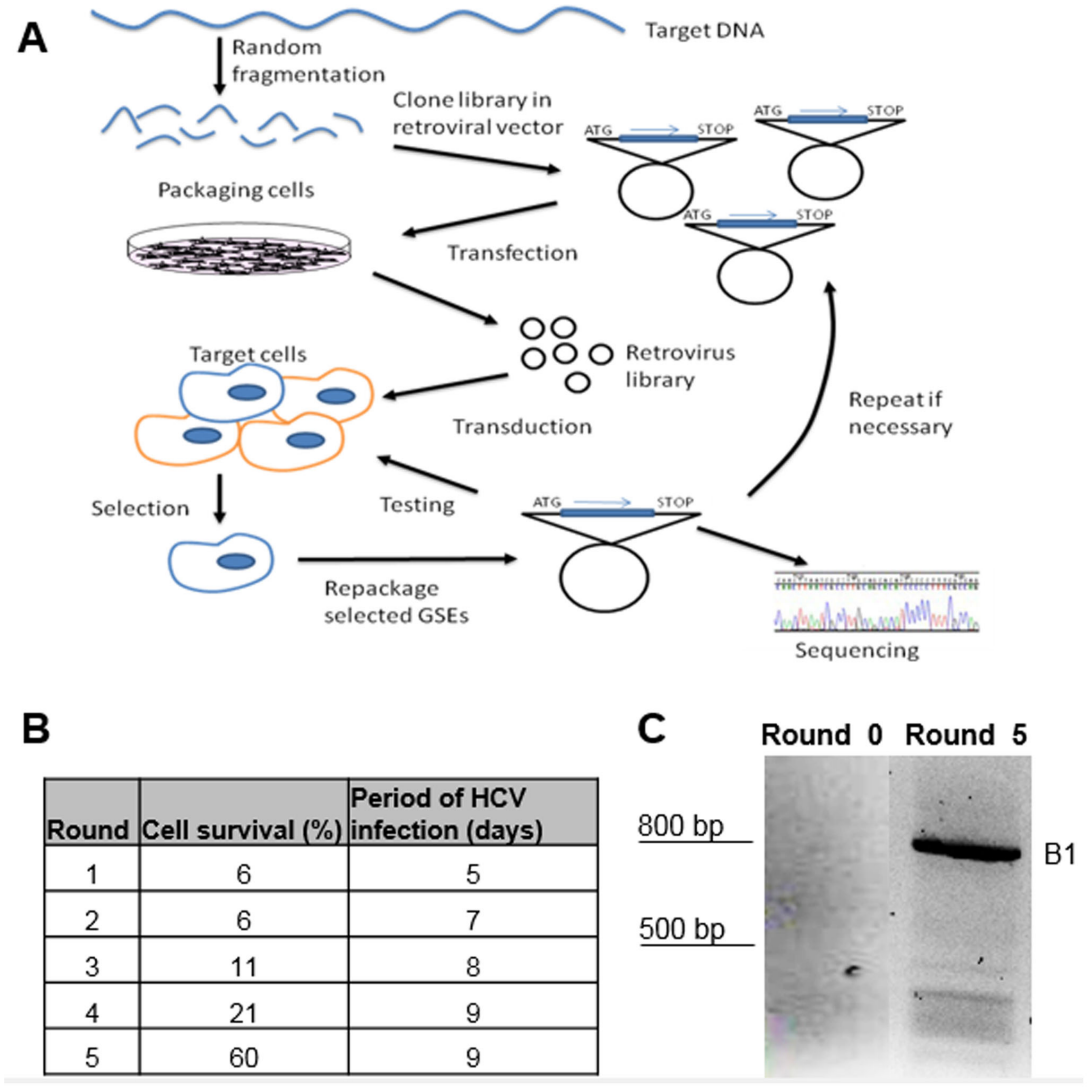
Overview of GSE selection. (**A**) GSE selection scheme. Randomly fragmented libraries were prepared by limited digestion of the HCV genome and fragments of 100–200 bp were cloned into the lentiviral vector pV1 and packaged into VSV-G-pseudotyped lentiviral particles. Library pseudoparticles were delivered into n4mBid cells such that each library fragment was expressed on average in at least 1000 cells. These cells were then infected with HCV and passaged for a period of 9 days or until the number of surviving cells was ≤10% of the initial cell population. The specific HCV inhibitor 2′C-MA was then added to rescue the surviving cells. Library fragments were repackaged by transfecting the surviving cells with plasmids encoding VSV-G and HIV gag-pol and formed the basis of subsequent rounds of selection. (**B**) Table depicting the number of cells that survived the HCV challenge as a percentage of the total number of seeded cells during consecutive rounds of selection. (**C**) Agarose gel image of cDNA inserts harvested from repackaged pseudoparticles after 5 rounds of selection.

A schematic of the approach used for GSE selection is presented in Figure 1. DNA fragments sized in the range 100–200 bp were obtained by DNaseI digestion of a plasmid encoding full-length Jc1 HCV [30]. These fragments were first polished to form blunt ends and then cloned into the lentiviral vector pV1 at the *Pme*I restriction site. pV1 is a minimal HIV-1 provirus lacking most HIV genes except for all necessary *cis* acting sequences such as Tat, Rev and Vpu ORF [31]. pV1 also lacks a Nef gene and in its place contains a cloning site for the insertion and expression of the cDNA of interest. cDNA inserts are expressed from the viral LTR. We chose the pV1 for library expression because viral particles can be repackaged from pV1-transduced cells when these cells are transfected with vectors encoding envelope glycoprotein and HIV gag-pol, facilitating the enrichment of selected GSEs [32].

In this study, the fragmented library was expressed as a C-terminal fusion to the transmembrane anchor of HCV NS4A (NS4Am) [33]. HCV replicates in complexes associated with the lipid raft membrane [34], [35] and HCV nonstructural (NS) proteins have been detected around lipid droplets in most HCV-permissive cells [36]. The attachment of the membrane-anchoring domain from a HCV NS protein to library fragments is designed to facilitate the interaction of the library fragments with viral/host factors located in the vicinity of the replication complexes, thus increasing the likelihood of successful identification of suppressor elements.

A randomly fragmented HCV genome was cloned into pV1 and this library was packaged into lentiviral particles via transfection of a packaging cell line. The lentiviral library was used to transduce the HCV reporter cell line n4mBid at a low multiplicity of infection (MOI) such that most transduced cells receive only one library fragment. The n4mBid cells contain a modified version of the pro-apoptotic protein Bid (mBid) and undergo apoptosis upon intracellular expression of HCV serine protease NS3-4A [37]. Two days post transduction, after the expression of the fragment library is established, these cells were infected with the Jc1 isolate of cell-culture-produced HCV (HCVcc) (MOI ∼1) [30] and maintained for a period of 9 days or until >90% of the cells succumbed to the HCV- induced cytopathic effect. The surviving cells were ‘cured’ of any ongoing HCV infection by exposure to the nucleoside analog 2′C-MA (1 µM) [38]. Library fragments present in the surviving cells were repackaged by transfection of these cells with plasmids encoding Gag-pol and VSV-G envelope protein. Repackaged lentiviruses were used in the subsequent round of selection (Figure 1A). In some cases, the repackaged viruses were amplified in 293T cells to increase the viral titer prior to the next round of selection.

In total, we screened a library containing an estimated 12, 000 individual fragments. For a library based on a fragmented retroviral HIV genome, the frequency of active perturbations (i.e. desirable GSE molecules) was estimated to be ∼1/6000 [17]. As shown in Figure 1B, the time taken to observe the cytopathic phenotype, as well as the percentage of surviving cells at the end of the selection, increased with each successive round of selection, suggesting that HCV-inhibitory fragments were being enriched. At the end of the fifth round of selection, library fragments were recovered from the repackaged lentivirus and the inserts were expressed as cDNA via RT-PCR. We observed significant enrichment of a fragment of ∼800 bp, and weaker enrichment several fragments with sizes below 500 bp (Figure 1C). The 800 bp fragment was recovered and named B1. Sequencing revealed that B1 is a NS4Am-eGFP fusion gene with a single-nucleotide insertion at the 3′ end of NS4Am just upstream of eGFP that results in a frame-shift of the eGFP gene (Figure S1). Although eGFP was not included in the fragment library by design, it was present in the vector that was used as the basis for cloning of the fragment library and was removed by restriction enzyme digestion prior to cloning. It is apparent that a small amount of the original eGFP-containing vector that additionally contained a frameshift-causing base insertion remained in the cloned library mixture. The frame-shifted eGFP gene yields a protein of 244 amino acids with a very high net positive charge of +43. The smaller enriched fragments shown in Figure 1C did not significantly inhibit Jc1 Gluc HCVcc (data not shown) and were not investigated further.

**Figure 2:**
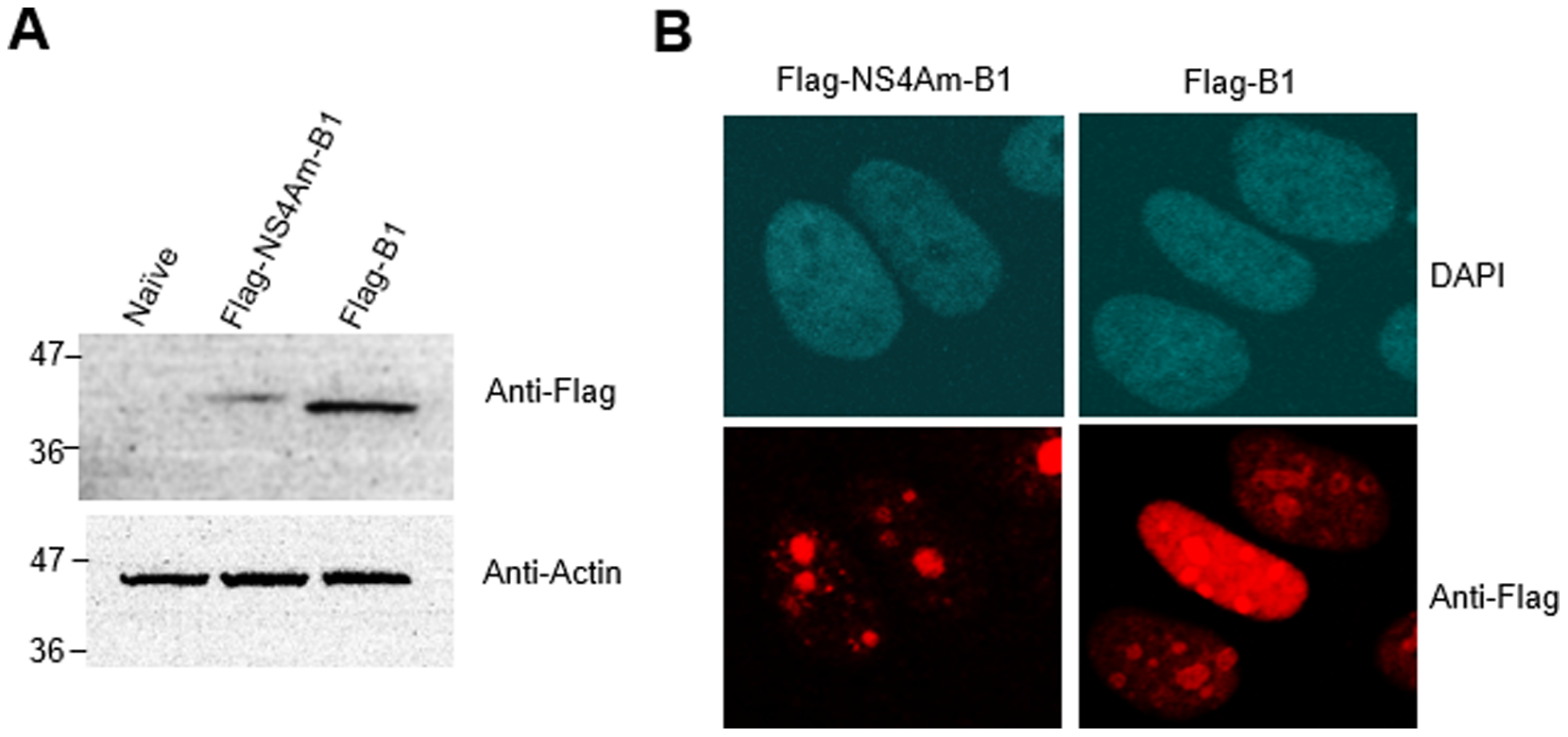
Expression of B1 in Huh-7.5 cells. (**A**) Western blot of Flag-NS4Am-B1 and Flag-B1. (**B**) Confocal microscopic images of Huh-7.5 cells stably expressing Flag-NS4Am-B1 or Flag-B1 after immunohistochemical staining with anti-flag antibody.

To confirm the expression of full-length B1 in Huh-7.5 cells, we constructed Flag-tagged B1 both with and without the NS4Am anchor (constructs 3 and 4 in Table 1; Figure 2A). The expression levels of the Flag-NS4Am-B1 appeared to be slightly lower than that of Flag-B1. We next determined the intracellular localization of both constructs using confocal microscopy. It was expected that Flag-NS4Am-B1 would be localized to the endoplasmic reticulum (ER) by the presence of the NS4Am membrane anchor. However, both Flag-NS4Am-B1 and Flag-B1 appeared to be predominantly localized to the cell nucleus and concentrated in nucleolar regions (Figure 2B). Flag-NS4Am-eGFP localized to an extra-nuclear space consistent with ER association (Figure S2A). Since no known nuclear localization signal is present in B1, we reason that the nuclear localization may derive from a tendency of B1 to associate with negatively charged genomic DNA during cell division.

**Table 1:** Plasmid constructs.

Number	Name	Molecular weight(kDa)	Expressionvectors
1	NS4Am-B1	30.6	pV1, pLenti6
2	B1	28.4	pV1, pLenti6
3	Flag-NS4Am-B1	33.3	pZsGreen
4	Flag-B1	31.1	pZsGreen
5	NS4Am-B2	17.8	pV1
6	NS4Am-B3	14.7	pV1
7	NS4Am-B4	13.2	pV1
8	NS4Am-B5	10.7	pV1
9	NS4Am-B6	6	pV1
10	+36GFP	28.4	pV1
11	NS4Am-eGFP	30.6	pv1

### Intracellular B1 Inhibits HCV Infection

**Figure 3:**
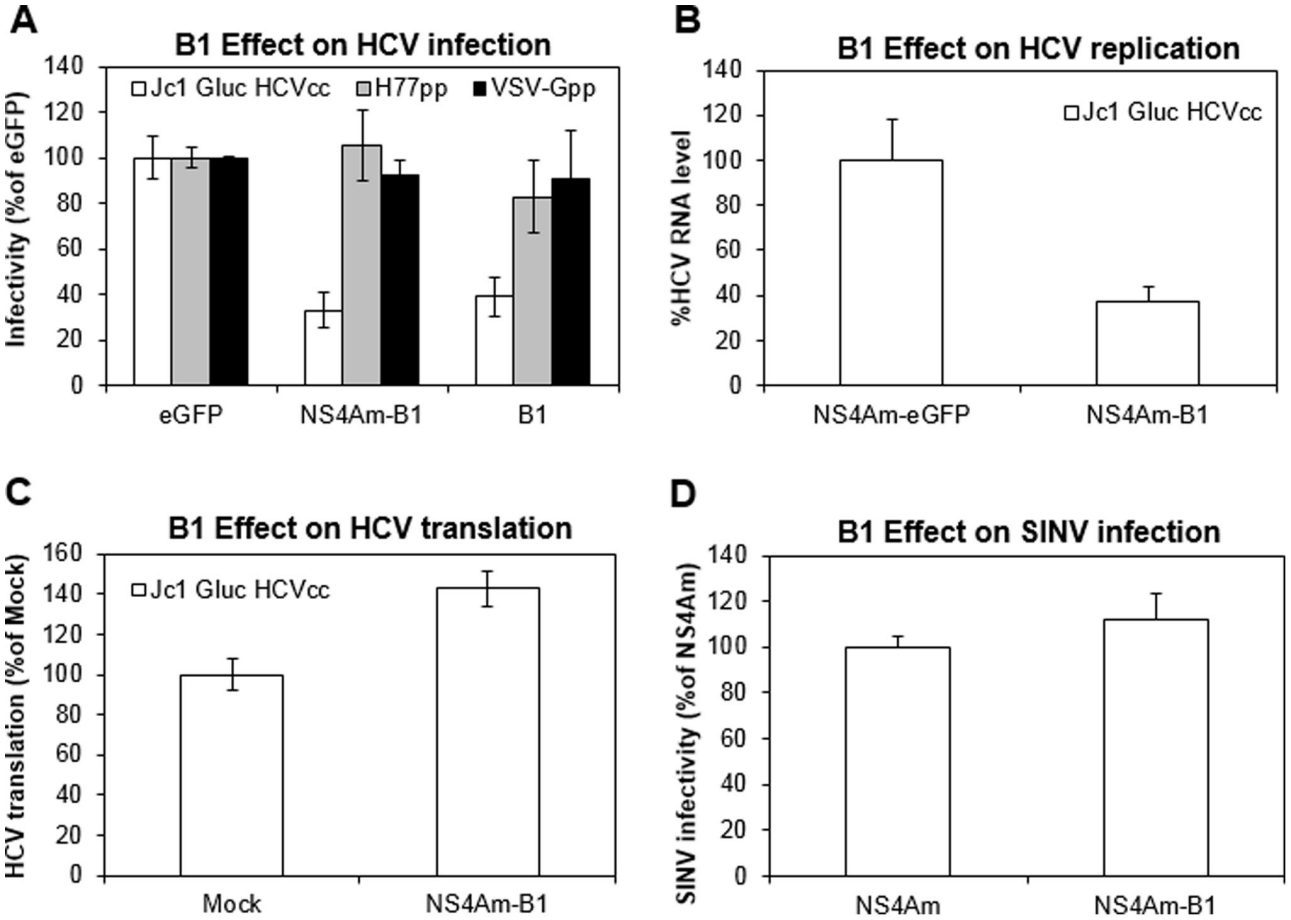
B1 inhibits HCV infection. (**A**) Cells expressing B1 or NS4Am-B1 are less permissive to HCV infection. Huh 7.5 cells were transduced with lentiviral pseudoparticles encoding eGFP, NS4Am-B1 or B1 at MOI ∼1 and selected for stable transductants prior to infection with JC1 Gluc HCVcc (MOI <0.1), H77 HCVpp (10-fold dilution) or VSV-Gpp (500-fold dilution) for 12 hours. The supernatant Gluc activity was quantified 24 hours later and used as indication of HCV infection level. (**B**) Cells expressing NS4Am-B1 suppress HCV replication. Huh-7.5 cells were transduced with lentiviral pseudoparticles encoding NS4Am-eGFP or NS4Am-B1 at MOI 4. 48 hours later, these cells were electroporated with RNA of full-length JC1 Gluc HCVcc. Intracellular HCV RNA levels were quantified 48 hours later using qRT-PCR. (**C**) B1 does not inhibit HCV translation. Naïve Huh-7.5 cells or cell populations expressing NS4Am-B1 (as in **B**) were electroporated with JC1 Gluc HCVcc. HCV translation was quantified by measuring the activity of secreted Gluc 5 hours later. (**D**) B1 does not inhibit SINV infection. BHK-J cells were transduced with lentiviral pseudoparticles encoding NS4Am-B1 or NS4Am alone at MOI ∼4. Two days later, cells were infected with SINV for 12 hours and thoroughly washed. Infectious SINV in the supernatant collected 48 hours later were quantified by plaque assay. All error bars represent the standard deviation of two independent experiments carried out in duplicate.

To assess the ability of B1 to inhibit HCV infection, the sequences of B1, NS4Am-B1 and eGFP were cloned into pLenti6 proviruses and packaged into pseudoparticles used to transduce naïve Huh-7.5 cells at MOI ∼1. After selection with blasticidin, the transduced cell populations were inoculated with Jc1 Gluc HCVcc [39], HIV-1 lentivirus pseudotyped with envelope protein from H77 HCV (H77pp) [40], or HIV-1 lentivirus pseudotyped with vesicular stomatitis virus glycoprotein (VSV-Gpp) [41]. As shown in Figure 3A, cells expressing either NS4Am-B1 or B1 exhibited reduced levels of Jc1 Gluc HCVcc [30] when compared to those expressing eGFP, confirming the ability of B1 to inhibit HCV infection. B1 did not detectably inhibit viral entry in cells inoculated with pseudotyped lentiviruses regardless of the envelope protein. The pseudotyped lentiviruses are able to enter cells but are unable to replicate. These results suggest that B1 does not affect the entry step of HCV infection or lentiviral transduction.

Since B1 appears to predominantly localize to the nucleus, and some nuclear factors are required for HCV replication [42]–[44], we next considered whether B1 inhibits HCV replication. Cell populations expressing NS4Am-eGFP or NS4Am-B1 were electroporated with RNA encoding either full-length Jc1 Gluc HCVcc [30]. The amount of intracellular HCV RNA was quantified 48 hours post electroporation by qRT-PCR. Similar levels of inhibition were observed in cells electroporated with Jc1 Gluc HCVcc [30] (Figure 3B) and in cells infected with the same virus (Figure 3A). No inhibition of HCV H77 replicon was observed (data not shown), suggesting that the effect of B1 may be limited to genotype 2 HCV.

Next, we considered whether B1 might inhibit the translation/processing of HCV because 1) B1 was previously found to strongly associate with mRNA due to its high positive charge [45] and 2) B1 primarily localizes to the nucleus and HCV translation is potentially modulated by nuclear factors [42], [43]. Cell populations expressing NS4Am-GFP or NS4Am-B1 were transfected with RNA expressing the full length Jc1 Gluc HCVcc [30].HCV IRES-mediated translation was quantified based on reporter activity measured at 5 hours post transfection (prior to the onset of replication [46]). No inhibition of Jc1 Gluc HCVcc translation was observed in cells expressing NS4Am-B1 (Figure 3C), indicating that B1 does not affect IRES-mediated translation.

B1 does not affect lentiviral transduction (Figure 3A), suggesting that B1 likely does not upregulate non-specific antiviral response mechanisms such as the interferon pathway or the unfolded protein response [47], [48]. To confirm that B1 does not activate the innate antiviral machinery, we determined the ability of B1 to inhibit the infection of Sindbis Virus (SINV), a closely related positive sense RNA virus belonging to the *Alphavirus* genus [49]. No inhibition of SINV was observed in cells expressing B1 (Figure 3D), supporting our hypothesis that SINV likely does not activate general cellular antiviral pathways.

### Shortened Positively Charged Forms of B1 Retain Anti-HCV Activity

**Figure 4:**
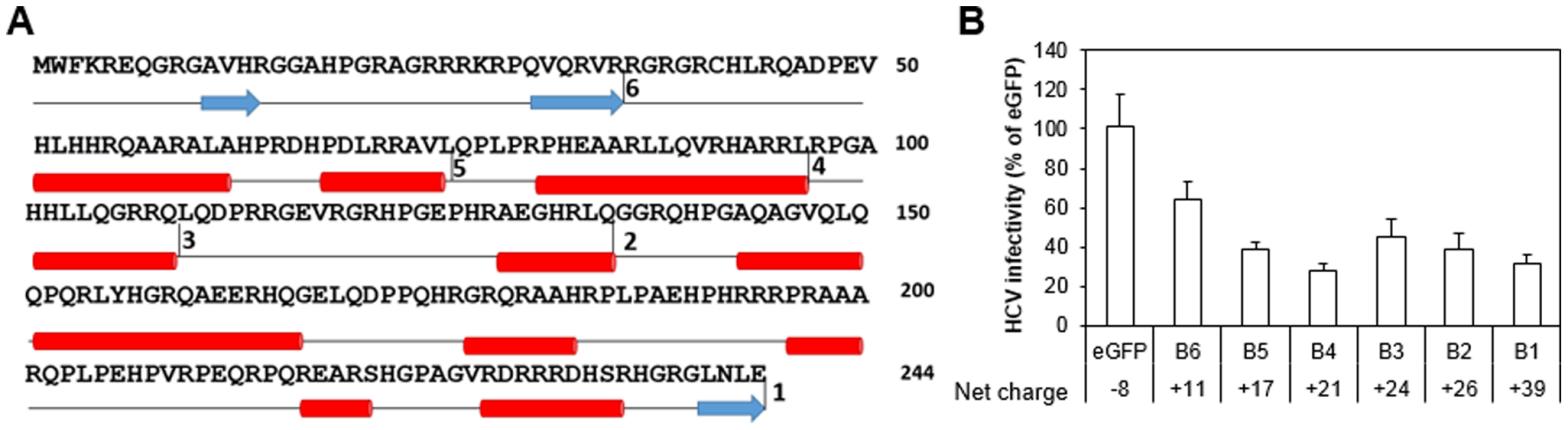
Shortened forms of B1 retain significant anti-HCV activity. (**A**) B1 was progressively truncated from the C-terminus to form the constructs B2– B6 without the disruption of secondary structural motifs predicted by the GOR4 algorithm. (**B**) Anti-HCV activity of constructs B1– B6. Huh-7.5 cells were transduced with lentiviral pseudoparticles encoding the indicated constructs at MOI ∼4. 48 hours later, these cells were infected overnight with JC1 Gluc HCVcc (MOI <0.1). Supernatant Gluc activities were quantified 48 hours later and used as indication of HCV infection level. Error bars represent the standard deviation of two independent experiments carried out in duplicate.

Next, we sought to determine whether a specific domain/region in B1 is responsible for the anti-HCV activity. B1 was progressively truncated at the C-terminus to form the shortened versions named B2– B6 (Figure 4A). Truncation sites were chosen such that they do not disrupt secondary structural motifs predicted by the GOR4 algorithm [50]. These shortened B1 fragments were cloned into the pV1 lentiviral vector and packaged into lentiviruses. Huh-7.5 cell populations expressing the shortened B1 fragments were challenged with Jc1 Gluc HCVcc at MOI <0.1 and the HCV infection levels were quantified 3 days post infection. All shortened B1 constructs appeared to inhibit HCV infection to a level comparable to the full-length B1 (Figure 4B), indicating that no specific region/domain in B1 is singly responsible for the anti-HCV activity. Fragments with a positive charge above 17 all suppressed HCV activity by >50%, while B6, the shortest fragment with a lower predicted charge of +11, exhibited slightly reduced anti-HCV activity. The negative control protein eGFP, with a predicted net charge of −8, showed no anti-HCV activity.

## Discussion

In this work we report the development of a selection method for the identification of genetic suppressor elements (GSEs) of HCV and characterize the anti-HCV properties of a highly enriched GSE from our application of the selection. Despite recent advances in direct acting antivirals (DAAs) against HCV, there remains a critical need for new HCV inhibitors with novel mechanisms of antiviral action. The selection method we developed utilizes a cell-death reporter hepatoma cell line for HCV previously developed in our laboratory, n4mBid [37]. A fragmented HCV genomic library is delivered to n4mBid cells via lentiviral transduction. Transduced cells are subsequently challenged with cell-culture-produced HCV (HCVcc) [30]. The surviving cells are pooled and the enriched genetic elements from these cells are harvested, repackaged, and used in subsequent rounds of selection/enrichment.

After 5 rounds of selection, we identified B1, a 244 amino acid protein derived from a rare frameshift-causing single-base insertion present immediately upstream of an enhanced GFP (eGFP) gene, a filler used during library cloning. B1 is highly positively charged and has been found to possesses the ability to penetrate and deliver biomolecular cargo into cells [45]. The eGFP sequence was not intended to be a part of the original library. It is even more unexpected that B1 derives from a single nucleotide insertion in the primer region upstream of eGFP (Figure S1). We showed that B1 is expressed as a full-length protein in Huh-7.5 cells (Figure 2A. Surprisingly, fluorescence microscopic images showed that B1 appeared to be almost exclusively localized in the nucleus even when fused to the NS4A membrane anchor (NS4Am) (Figure 2B). It is possible that an undetectable amount of B1 remains in the cytosol. Despite the nuclear localization, both unmodified B1 and NS4Am-B1 exhibit a similar anti-HCV potency (Figure 3A). Further characterization revealed that B1 does not affect HCV entry (Figure 3A) or virus production (data not shown) but specifically inhibits the replication step of the HCV life cycle (Figure 3B). Some nuclear factors such as heterologous nuclear ribonucleoprotein L (hnRNP L) [42] and the nuclear La protein [43] have been demonstrated to affect HCV replication by upregulating HCV translation. However, B1 does not appear to inhibit IRES-mediated HCV translation (Figure 3C). Recently, it was shown that nuclear translocation of HCV NS5A regulates HCV replication [51]. The antiviral mechanism of action of B1 remains enigmatic. We speculate that B1’s ability to inhibit HCV replication may be attributable to the presence of a small amount of B1 in the cytosol and/or interaction of B1 with nuclear factors required for HCV replication.

To shed light on B1’s mechanism of anti-HCV action, a series of shortened B1 constructs with progressive deletions at the C-terminus were generated. We reasoned that, if the high net positive charge of B1 was not a major factor contributing to the anti-HCV activity and the inhibition is due instead to a specific structural motif or region in B1, then once the motif was removed from the construct, anti-HCV activity should be completely abrogated. We generated shortened forms of B1 with predicted net charges ranging from +11 to +39 and showed that all these fragments, including the shortest fragment (35 amino acids with a predicted charge +11), retained most, if not all, of the anti-HCV activity of the parent construct B1 (Figure 4B). This result suggests that the high net positive charge, rather than a specific motif, could be responsible for the anti-HCV activity of B1.

While positively charged proteins are relatively uncommon in nature, many are expressed in mammalian cells [52]. Previously, Liu and coworkers synthesized a series supercharged variants of GFP and showed that these molecules can effectively transduce mammalian cells [53]. We tested the ability of one of these supercharged proteins, +36 GFP, to inhibit HCV infection. +36GFP possesses a net positive charge of +39 and as such is comparable to B1 in overall charge. Fluorescence imaging of intracellularly expressed +36GFP indicated that it was predominantly localized in the cell nucleus (Figure S2A), similar to B1 (Figure 2B). However, even after high-MOI transduction (up to MOI ∼10), only a small percentage (∼20%) of the transduced cells showed detectable +36GFP expression (Figure S3B). Perhaps for this reason, no HCV inhibition was observed in cells transduced with a vector expressing +36GFP (Figure S2C). Interestingly, in contrast, cells exposed to purified recombinant B1 and +36GFP at the time of infection showed a reversed phenotype. Purified B1 added extracellularly to cells at the same time of HCV or lentivirus did not inhibit infection, while extracellularly added +36GFP strongly inhibited HCV infection and partially inhibited VSV-Gpp infection (Figure S3). The discrepancy between the anti-HCV profiles of B1 and +36GFP on intracellular expression vs. after extracellular exposure may derive in part from a different direct action of the two molecules on extracellular virus and in part from the different intracellular distributions of the molecules produced from the two delivery methods. Intracellularly expressed B1 (Figure 2B) and +36GFP (Figure S2A) both localize to the nucleus. In contrast, extracellularly delivered B1 exhibits a punctate distribution on the paranuclear region of cells, while extracellularly delivered +36GFP accumulates evenly in a large number of tiny, endosomal compartments [45].

Taken at face value, the anti-HCV activity of B1 is comparable to the antiviral effects of defensins. Defensins are short (less than 100 amino acids), positively charged peptides that contain six cysteine residues [54]. Defensins are broad-spectrum antimicrobials and have been shown to be active against bacteria, fungi and some enveloped viruses [55]–[59]. The antiviral activity of defensins is largely attributed to the molecules’ positive charge [56]. However, defensins act directly on extracellular viruses while B1 inhibits intracellular HCV replication (Figure 3B). Exposing cells to purified B1 protein prior to or during HCV infection did not yield HCV inhibition (Figure S3). Extracellularly delivered B1 exhibited a punctate distribution in the paranuclear region of cells [29], comparable to the distribution of extracellularly delivered +36GFP [60]. However, intracellularly expressed B1 appeared to exclusively localize to the nucleus (Figure 2B). The difference in subcellular localization of B1 may be critical to the molecule’s ability to inhibit HCV replication. Human alpha defensins 1–3 were found to act intracellularly to inhibit PKC signaling, which results in inhibition of HIV infection [61]. However, no inhibition of pseudotyped lentivirus was observed in cells expressing B1 (Figure 3A), suggesting that B1 does not significantly inhibit PKC signaling and B1 does not function like a defensin in these cells. In contrast, extracellularly delivered +36GFP did significantly inhibit HCV and pseudotyped lentivirus infection (Figure S3), implying that unlike B1, recombinant +36GFP may possess some defensin-like antiviral activity.

In conclusion we developed an *in vitro* method for selecting GSEs of HCV. Application of this selection led to the unexpected identification of a highly positively charged protein, B1, which inhibits HCV replication. Although B1 does not exist in nature, and cannot be generated by a frame shift of any natural proteins, it is potentially usefully useful for biomedical applications due to 1) its ability to inhibit HCV replication and 2) mediate protein transduction [45]. Minimization studies showed that B1’s anti-HCV activity is not entirely attributable to any specific region of the molecule, while shortened positively charged variants of B1 all retained some anti-HCV activity, suggesting a general role of a high net positive charge in the antiviral activity. Follow-up studies showed that another highly positively charged protein, +36GFP, also possesses anti-HCV activity, although these preliminary studies point to a potentially different antiviral mode of action of +36GFP relative to B1 when added extracellularly to cells at the time of infection. Proteins with high net positive charge represent a novel class of anti-HCV molecules. In addition, the GSE selection approach described in this report can be applied to other protein/peptide libraries in the continued search for new HCV-inhibitory molecules.

## Materials and Methods

### Reagents, Bacterial Strains and Cell Lines

TZM-bl cells were obtained from Dr. John C. Kappes, Dr. Xiaoyun Wu and Tranzyme Inc. through the NIH AIDS Reagent Program, Division of AIDS, NIAID [62]. Huh-7 and Huh-7.5 cells [63] were obtained from Prof. Charles Rice (Rockefeller University). 293T cells were purchased from Life Technologies (Grand Island, NY).

All cell lines were cultured in complete growth medium (Dulbecco’s Modified Eagle Medium (DMEM) containing 4500 mg/L glucose, 4.0 mM L- Glutamine, and 110 mg/L sodium pyruvate (Thermo Scientific HyClone, Logan, UT) supplemented with 10% fetal bovine serum (Atlanta Biologicals, Lawrenceville, GA) and 1X non- essential amino acids (Thermo Scientific HyClone, Logan, UT)). *Escherichia coli* strains DH5α and Stbl3 (Life technologies) were used for recombinant DNA cloning.

Biolux Gaussia Luciferase Assay Kit was purchased from New England Biolabs (Ipswich, MA). Dulbecco's Phosphate Buffered Saline (DPBS) was purchased from Thermo Scientific HyClone. OptiMEM and Lipofectamine 2000 were purchased from Life Technologies. Luria- Bertani (LB) broth, Mirus TransIT LT1 transfection reagent and ampicillin were obtained from Thermo Fisher Scientific. 0.25% trypsin- EDTA was from VWR International. All restriction enzymes, nucleases and polymerase were purchased from New England Biolabs.

### Fragment Library Preparation

Plasmid DNA encoding full-length Jc1-NFlag2 HCVcc [30] was digested with DNaseI at 16°C for minutes to obtain an average fragments size between 100–200 bp. The digested DNA were extracted by phenol-chloroform following standard protocol, sequentially treated with Mung Bean Nuclease, T4 DNA polymerase and Klenow Fragments to form blunt ends, and phosphorylated using T4 Polynucletide Kinase. Blunted fragments were inserted into the pV1 lentiviral vector downstream of NS4A membrane anchoring domain (NS4Am). pV1 is a minimal HIV-1 provirus lacking most of the HIV genes except for essential *cis*-acting elements and the tat, rev and vpu genes [64]. The vector backbone was prepared by digesting pV1-NS4Am-eGFP with PmeI to remove the eGFP insert and dephosphorylated using Antarctic Phosphotase. The ligation mixture was electroporated into *E. coli* DH5α. Colonies (∼12,000) were pooled and cultured, and the plasmid DNA library (Lib4) was isolated.

### Lentiviral Pseudoparticle Production and Titering

Lib4-expressing pseudoparticles (Lib4pp) were generated based on a procedure described previously [65]. Briefly, 293T cells were transfected with a 2∶2∶1 mass ratio of plasmids encoding HIV gag-pol, vesicular stomatitis virus glycoprotein (VSV-G), and Lib4 using TransIT-LT1 transfection reagent [66]. Cell supernatants containing VSV-G-coated Lib4pp were collected 48 hours later, filtered and stored at −80°C until use. Collected Lib4pp were titered in TZM-bl indicator cells. TZM-bl cells were inoculated with serial dilutions of Lib4pp (10^−1^–10^−6^). 48 hours later, cells were washed with DPBS, fixed in PBS/2% paraformaldehyde (PFA) and stained with X-Gal (VWR International). The TCID_50_ of the Lib4pp in TZM-bl was calculated using the method of Reed and Meunch [67]. The titer determined in TZM-bl cells was correlated to that in Huh-7.5 cells by comparing the infectivity of GFP-expressing pseudoparticles (GFPpp) in both cell types. GFPpp was generated using the same protocol but with the replacement of Lib4-expressing plasmid with pTRIP-GFP [68]. Huh-7.5 and TZM-bl cells were inoculated with serial dilutions of GFPpp (10^−1^–10^−6^). 48 hours later, Huh-7.5 cells were collected and analyzed via flow cytometry (FACScan flow cytometer, BD Biosciences) while infection of TZM-bl cells was analyzed as described above. The infectivity of the pseudoparticles was determined based on infection at low multiplicity of infection (MOI) and correlated between these two cell lines.

Lentivirus pseudotyped with envelope proteins from HCV H77 (H77pp) [40] and VSV-G (VSV-Gpp) [41] were generated from 293T cells as previously described [65]. Both H77pp and VSV-Gpp harbor the pTRIP-Gluc reporter provirus. Briefly, 293T cells were transfected with a 1∶4∶1 mass ratio of HIV gag-pol, Gluc, and H77/VSV envelope plasmids using TransIT-LT1 transfection reagent [66]. Cell supernatants containing H77pp or VSV-Gpp were collected 48 hours later, filtered and stored at −80°C until use.

### Library Selection

For each round of selection, 2.1×10^7^ n4mBid cells were transduced with Lib4pp or repackaged lentiviral pseudoparticles harboring enriched fragments at MOI ∼0.5. This allowed for the expression of each theoretical library fragment in at least 1000 cells on average. Cells were cultured for 48 hours post transduction to allow for fragment expression. Next, these cells were challenged with Jc1 HCVcc at MOI ∼1, and cultured for 9 days or until <10% of the initial cell population remained viable. Surviving cells were ‘rescued’ from HCV infection by exposure to the HCV polymerase inhibitor 2′C-MA (1 µM) for 72 hours. Library fragments in the surviving cell population were recovered by transfecting these cells with plasmids encoding HIV gag-pol and VSV-G using Fugene-6 transfection reagent (Promega). The choice of the Fugene-6 reagent here derives from its low cytotoxicity in these cells. Cell supernatants containing VSV-G-coated pseudoparticles of repackegd fragments were collected 48 hours later, filtered and stored at −80°C. Since Huh-7.5 cells are difficult to transfect, lentiviral pseudoparticles repackaged from Huh-7.5 cells needed to be amplified prior to the subsequent round of selection. Briefly, 293T cells were first transduced with ten-fold diluted repackaged pseudoparticles. 48 hours later, these cells were transfected with plasmids encoding HIV gag-pol and VSV-G using Trans-IT LT1 transfection reagent (Mirus). Cell supernatants containing high titers of repackaged Lib4pp were collected 48 hours later, filtered and stored at −80°C. This amplification step typically increases the lentiviral titer by ∼100-fold. Recovered supernatants were titered in TZM-bl cells as described above and used for subsequent rounds of infection. After the final round of selection, viral RNA were isolated using RNeasy Mini Kit (Qiagen) and library fragment cDNA were amplified by RT-PCR (ImProm-II, Promega) using the primers Lib4-F (5′-ACG GCC TCT AGA ATG AGC-3′) and Lib4-R (5′-AGT GGC TAA GTC TAC AGC TG-3′). Amplified fragments were analyzed on a 1.5% agarose gel, as shown in Figure 1C.

### Immunofluorescence Imaging and Western Blots

Huh-7.5 cells were seeded into wells of a glass chamber slide before being transduced with lentiviral pseudoparticles expressing Flag-tagged NS4Am-B1 and B1 (Constructs #3-4, Table 1). Two days post transduction, cells were fixed by incubation with PBS/2% PFA for 30 minutes at 4°C, stained with mouse anti-Flag primary antibody (Genscript) and Alexa-Fluor 568-conjugated goat anti-mouse secondary antibody (Life Technologies), treated with antifade reagent (Promega) overnight and imaged using a Zeiss 510 Meta NLO Multiphoton microscope (Carl Zeiss Microscopy).

For Western blots, cells were lysed with Renilla luciferase assay lysis buffer (Promega) two days post transduction. Cell lysates were combined with an equal volume of 2X SDS loading buffer and incubated at 95°C for five minutes. Samples were resolved using SDS-PAGE (12% acrylamide) and transferred to a PVDF membrane. After transfer, the membranes were sequentially incubated with mouse anti-Flag primary antibody (Genscript) and HRP-conjugated donkey anti-mouse secondary antibody (Jackson ImmunoResearch), developed using West Pico Chemiluminescent HRP substrate (Pierce) and visualized in a ChemiDoc-It (UVP) chemiluminescence imager.

### HCV Infection Assay

To evaluate the anti-HCV activity of B1, Huh-7.5 cells were transduced with pLenti6-GFP/B1/NS4Am-B1pseudoparticles at low (<0.5) MOI, selected with blasticidin (10 µg/mL) for 8 days and inoculated with Jc1 Gluc HCVcc (MOI <0.1), H77pp (10-fold diluted supernatant) or VSV-Gpp (500-fold diluted supernatant) for 12 hours. The cells were thoroughly washed with complete growth medium to remove residual Gluc protein and HCV infectivity in each cell population was quantified 24 hours later by measurement of the activity of the secreted Gluc reporter protein in the supernatant.

### HCV Replication and Translation Assay

Cells expressing eGFP or B1 were electroporated with the genomic RNA of full-length Jc1 Gluc HCVcc [30]. Five hours post electroporation, HCV translation was quantified by measurement of reporter Gluc activity in the cell supernatants. Forty eight hours post electroporation, cells were harvested and intracellular HCV RNA levels were quantified by qRT-PCR as described previously [65].

### SINV Production and Infection Assay

Infectious SINV was generated and titered in BHK-J cells as described previously [69]. Briefly, 1×10^7^ BHK-J cells were suspended in 400 µl DPBS and electroporated with 3 µg *in vitro*-transcribed SINV Toto1101 RNA [49] using an ECM 830 electroporator (Harvard Apparatus) programmed with the following settings: 750 V, 99-µs pulse length, 5 pulses, 1.1-s interval. Cell supernatants were collected 24 hours post electroporation, filtered and stored at −80°C. SINV titers were determined by exposing BHK-J cells to ten-fold serial dilutions of virus prior to quantification via a plaque assay 24 hours later.

To evaluate the ability of B1 to inhibit SINV, BHK-J cells were transduced with pV1-NS4Am-eGFP/B1 (Table 1, construct 1)-expressing pseudoparticles at MOI ∼4. Forty eight hours later, these cells were infected overnight with SINV at MOI ∼10. Cell supernatants were collected 36 hours later upon cell lysis and the amount of infectious SINV in each supernatant was quantified via plaque assay.

## Supporting Information

Figure S1 **B1 nucleotide and amino acid sequences.** Image showing comparative nucleotide (black) and amino acid (blue) sequences of eGFP (above) and B1 (below). Insertion which lead to the generation of B1 is shown in red. Nucleotides encoding NS4Am amino acids are highlighted in grey.
(TIF)

Figure S2 **Intracellularly expressed +36GFP does not inhibit HCV infection.** Huh-7 cells were transduced with lentiviral pseudoparticles expressing the indicated constructs at MOI ∼10. It was noted that using Huh-7 cells resulted in better expression of the +36GFP construct. Two days later, cells were exposed to HCVcc at MOI <0.1 for 12 hours. Cell supernatants were collected 48 hours post infection. At this time NS4Am-eGFP and +36GFP expression were visualized with a fluorescence microscope (**A**). After imaging, cells were trypsinized and the percentage of +36GFP-expressing cells was quantified via flow cytometry (**B**). HCV infection levels were quantified based on secreted Gluc levels in the collected supernatants (**C**). Error bars represent the standard deviation of two independent experiments carried out in duplicate.
(TIF)

Figure S3 **Purified B1 protein does not inhibit HCV infection.** Huh-7.5 cells were inoculated with Jc1 Gluc HCVcc **(A)** or H77pp/VSV-Gpp **(B)** in the presence of the indicated concentrations of GFP-L-B1, +36GFP or NH_4_Cl for 12 hours. Subsequently, cells were washed and replenished with media containing the same amounts of protein/drug. Levels of infection in each cell population were quantified 48 hours later via measurement of the secreted Gluc levels. Error bars represent the standard deviation of two independent experiments carried out in duplicate.
(TIF)
