# Supplementary figures and images for: A Screen for Genetic Suppressor Elements of Hepatitis C Virus Identifies a Supercharged Protein Inhibitor of Viral Replication
Source: PLoS One. 2013 Dec 31;8(12):e84022. doi: 10.1371/journal.pone.0084022 (PMC3877138; doi:10.1371/journal.pone.0084022)

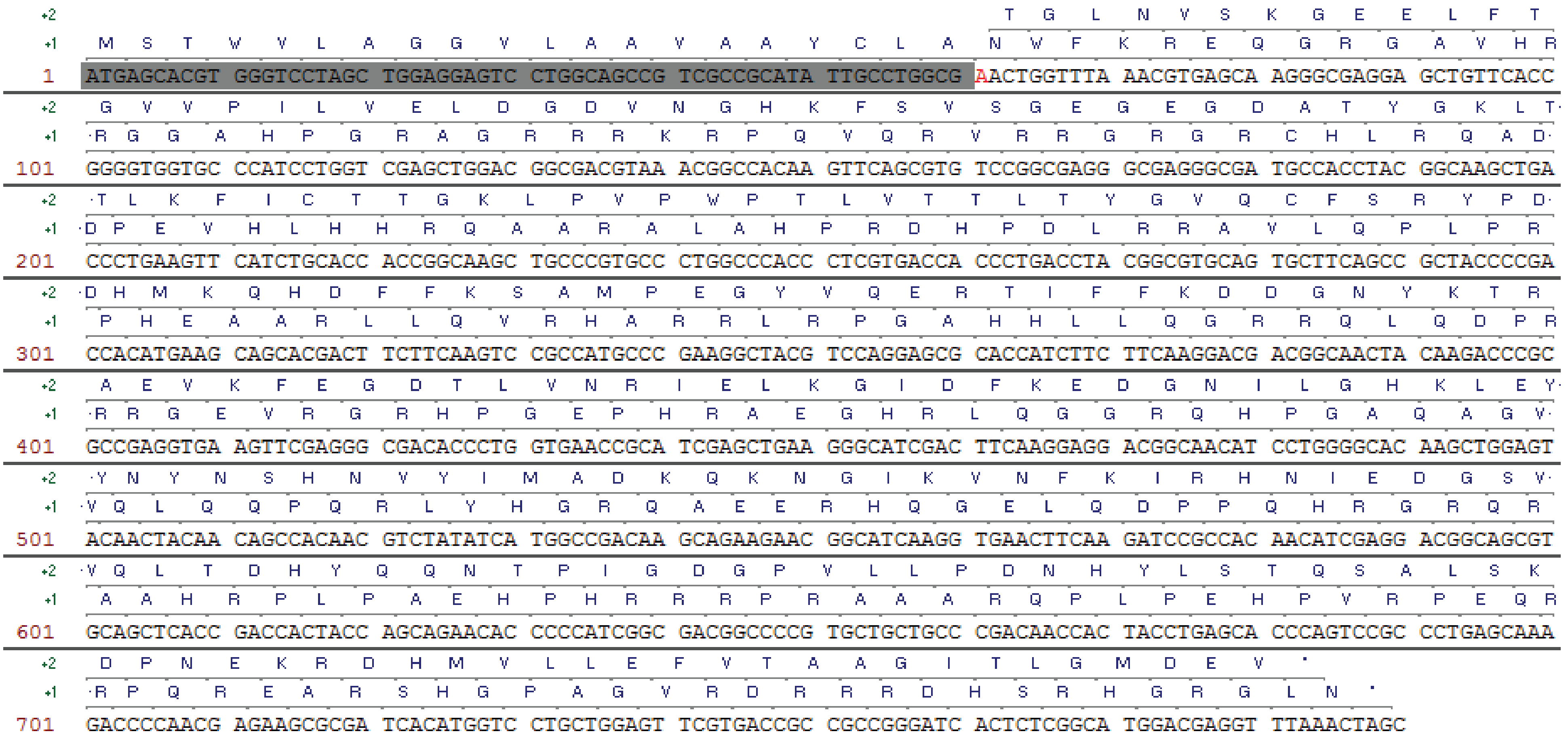

Supplement: Figure S1 — B1 nucleotide and amino acid sequences. Image showing comparative nucleotide (black) and amino acid (blue) sequences of eGFP (above) and B1 (below). Insertion which lead to the generation of B1 is shown in red. Nucleotides encoding NS4Am amino acids are highlighted in grey. (TIF) [file pone.0084022.s001.tif]

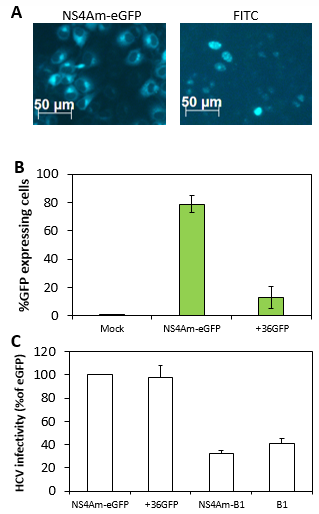

Supplement: Figure S2 — Intracellularly expressed +36GFP does not inhibit HCV infection. Huh-7 cells were transduced with lentiviral pseudoparticles expressing the indicated constructs at MOI ∼10. It was noted that using Huh-7 cells resulted in better expression of the +36GFP construct. Two days later, cells were exposed to HCVcc at MOI <0.1 for 12 hours. Cell supernatants were collected 48 hours post infection. At this time NS4Am-eGFP and +36GFP expression were visualized with a fluorescence microscope (A). After imaging, cells were trypsinized and the percentage of +36GFP-expressing cells was quantified via flow cytometry (B). HCV infection levels were quantified based on secreted Gluc levels in the collected supernatants (C). Error bars represent the standard deviation of two independent experiments carried out in duplicate. (TIF) [file pone.0084022.s002.tif]

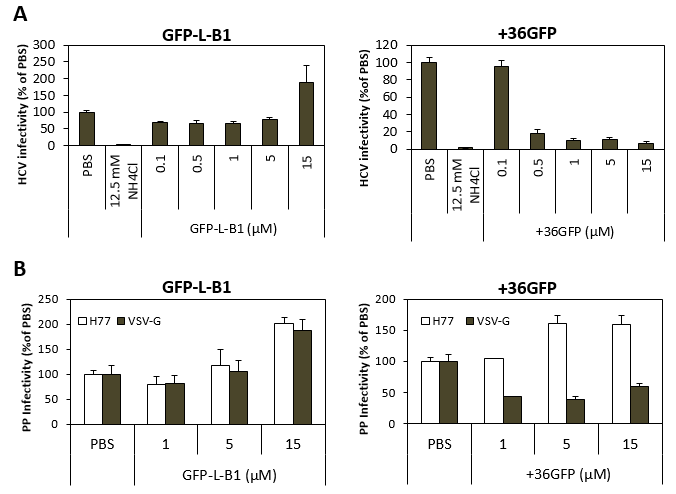

Supplement: Figure S3 — Purified B1 protein does not inhibit HCV infection. Huh-7.5 cells were inoculated with Jc1 Gluc HCVcc (A) or H77pp/VSV-Gpp (B) in the presence of the indicated concentrations of GFP-L-B1, +36GFP or NH4Cl for 12 hours. Subsequently, cells were washed and replenished with media containing the same amounts of protein/drug. Levels of infection in each cell population were quantified 48 hours later via measurement of the secreted Gluc levels. Error bars represent the standard deviation of two independent experiments carried out in duplicate. (TIF) [file pone.0084022.s003.tif]
